# Size-Exclusion Chromatography: A Path to Higher Yield and Reproducibility Compared to Sucrose Cushion Ultracentrifugation for Extracellular Vesicle Isolation in Multiple Myeloma

**DOI:** 10.3390/ijms25158496

**Published:** 2024-08-03

**Authors:** Madalena Grenhas, Raquel Lopes, Bruna Velosa Ferreira, Filipa Barahona, Cristina João, Emilie Arnault Carneiro

**Affiliations:** 1 Myeloma Lymphoma Research Group, Champalimaud Experimental Clinical Research Programme, Champalimaud Foundation, 1400-038 Lisbon, Portugal; madalena.grenhas@research.fchampalimaud.org (M.G.); raquel.lopes@research.fchampalimaud.org (R.L.); bruna.ferreira@research.fchampalimaud.org (B.V.F.); filipa.barahona@research.fchampalimaud.org (F.B.); cristina.joao@research.fchampalimaud.org (C.J.); 2 Faculty of Medicine, University of Lisbon, 1649-028 Lisbon, Portugal; 3 Hemato-Oncology Department, Champalimaud Foundation, 1400-038 Lisbon, Portugal; 4 Faculty of Medical Sciences, NOVA Medical School, 1169-056 Lisbon, Portugal

**Keywords:** extracellular vesicles, extracellular vesicle isolation methods, sucrose cushion ultracentrifugation, ultrafiltration, size-exclusion chromatography, multiple myeloma, myeloma cell lines, plasma, newly diagnosed multiple myeloma

## Abstract

Small extracellular vesicles (EVs) play a pivotal role in intercellular communication across various physiological and pathological contexts. Despite their growing significance as disease biomarkers and therapeutic targets in biomedical research, the lack of reliable isolation techniques remains challenging. This study characterizes vesicles that were isolated from conditioned culture media (CCM) sourced from three myeloma cell lines (MM.1S, ANBL-6, and ALMC-1), and from the plasma of healthy donors and multiple myeloma patients. We compared the efficacy, reproducibility, and specificity of isolating small EVs using sucrose cushion ultracentrifugation (sUC) vs. ultrafiltration combined with size-exclusion chromatography (UF-SEC). Our results demonstrate that UF-SEC emerges as a more practical, efficient, and consistent method for EV isolation, outperforming sUC in the yield of EV recovery and exhibiting lower variability. Additionally, the comparison of EV characteristics among the three myeloma cell lines revealed distinct biomarker profiles. Finally, our results suggest that HBS associated with Tween 20 improves EV recovery and preservation over PBS. Standardization of small EV isolation methods is imperative, and our comparative evaluation represents a significant step toward achieving this goal.

## 1. Introduction

Extracellular vesicles (EVs) serve as vehicles in intercellular communication, mediating various physiological and pathological processes [1]. These lipid-bilayer particles, released by all cell types into the extracellular space, carry a rich variety of proteins, lipids, nucleic acids, and other bioactive molecules [2]. With remarkable heterogeneity in size, content, and cellular origin, EVs are classified into three main subtypes: exosomes (30–150 nm), released by exocytosis, microvesicles (100–1000 nm), and apoptotic bodies (50–5000 nm), with the latter two released by ectocytosis [3]. Consistent with the guidelines outlined in the Minimal Information for Studies of Extracellular Vesicles (MISEV) 2018 [4], we use the term ‘EV’ in the present article to encompass all lipid bilayer particles secreted by cells.

EVs are attracting a lot of attention because of their remarkable clinical relevance across various medical fields [5]. As interest in EVs grows, and given the complexity of their subpopulations, the need for more refined and standardized methodologies to accurately isolate and characterize these vesicles becomes imperative [6]. To date, several isolation techniques are available, either from biofluids or from cell culture supernatants, each having advantages and limitations [7]. To unlock the full potential of EVs, standardized EV isolation protocols that achieve high yields, great homogeneity, and purity, in a short time and at a low cost, are needed.

Ultracentrifugation (UC) is the most commonly used method, employing sequential centrifugations to eliminate cellular debris and larger vesicles [8]. In sucrose cushion UC (sUC), EVs are further separated by a 30% sucrose density cushion, equivalent to the density of EVs (1.10–1.19 g/mL) [9]. Despite the efficiency of this method, it requires substantial time, volume, and specialized equipment, and the elevated centrifuge forces involved can potentially damage vesicles [10].

Size-exclusion chromatography (SEC) uses a porous polymer matrix to collect several EV fractions (F) at pre-determined time intervals [11]. It offers cost-effectiveness and preserves vesicle structure and biological activity, providing a quicker alternative to sUC [12]. However, SEC is limited to small volumes and produces diluted EV samples. Ultrafiltration (UF) can concentrate samples before or after SEC to address this issue [13], and combining these size-based techniques is a strategy to enhance purity and yield [14]. Although SEC has shown significant advantages over other methods [15,16], direct comparative analyses are missing.

In this study, we provide a comprehensive comparison of sUC vs. UF combined with SEC (UF-SEC), assessing the efficiency, reproducibility, and specificity of EV isolation. Additionally, we explored various buffers to optimize sample recovery and preservation. Our study focused on EVs derived from human myeloma cell lines, namely MM.1S, ANBL-6, and ALMC-1, selected for their genetic resemblance to the malignant cells found in multiple myeloma (MM) patients [17]. We also analyzed EVs isolated from the plasma of healthy donors and MM patients. MM is a hematological cancer characterized by uncontrolled plasma cell proliferation and monoclonal immunoglobulin overproduction in the bone marrow. The significant role of EVs in MM regarding pathogenesis, disease progression [18], and drug resistance [19] has been well established. EVs have emerged as promising clinical biomarkers for early diagnosis [20], disease classification, and therapy monitoring [21], as well as even being discussed as potential therapeutic targets for the treatment of MM patients [22]. Although numerous MM cellular models are available, studies focusing on EVs derived from human myeloma cell lines remain scarce. Since each cell line secretes unique EV subpopulations with specific cargoes [23], further characterization is required to determine the individual specificities of EVs from each myeloma cell line, permitting a closer correlation between the patients’ disease and cellular models**.**

We show that EV isolation by UF-SEC outperforms sUC in yield and reproducibility, both from human plasma and conditioned cell culture media samples, allowing for the utilization of smaller amounts of initial biological material. Furthermore, we identify distinct characteristics among the three myeloma cell lines. With this research, we aim to provide valuable insights that will help reach standardization in EV studies while advancing the use of cellular models in MM.

## 2. Results

### 2.1. Characterization of Isolated EVs

#### 2.1.1. Particle and Protein Concentrations

**Figure 1:**
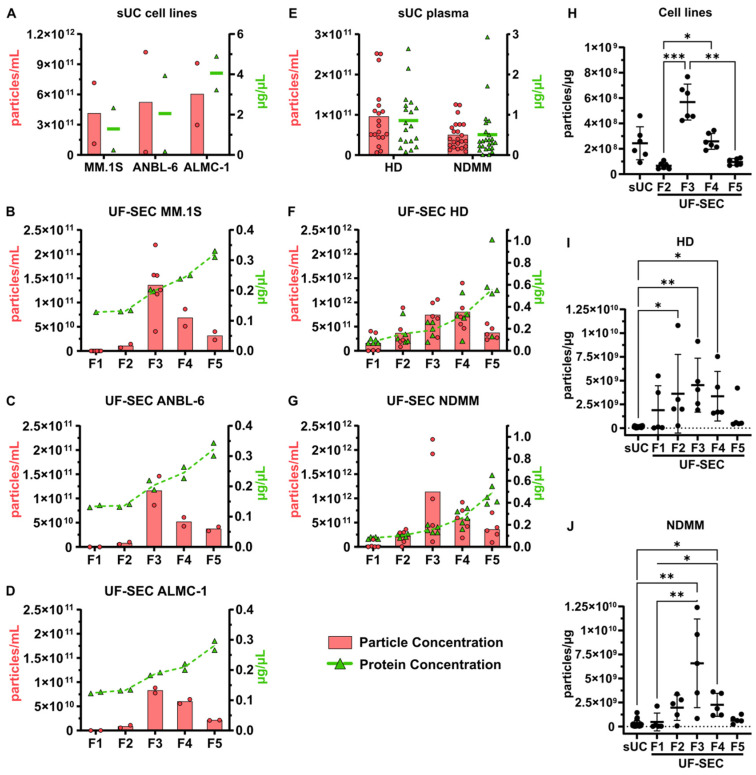
Particle and protein concentrations of isolated EV samples. EV isolation was performed using sUC (**A**,**E**) or UF-SEC (**B**–**D**,**F**,**G**). EVs were isolated either from conditioned culture medium (CCM) from three myeloma cell lines: MM1S ((**A**,**B**); *n* = 2 to 6); ANBL-6 ((**A**,**C**); *n* = 2); and ALMC-1 ((**A**,**D**); *n* = 2); or from human plasma samples, including healthy donors ((**E**,**F**); HD; *n* = 19 for sUC, *n* = 5 for UF-SEC) and newly diagnosed multiple myeloma patients ((**E**,**G**); NDMM; *n* = 24 for sUC, *n* = 5 for UF-SEC). Distinct particle and protein concentrations are observed between sUC and UF-SEC because different initial sample volumes were used (see methods). In UF-SEC, fraction 1 (F1) to fraction 5 (F5) were sequentially collected. Individual and mean values for particle (red dots and bars, respectively) and protein (green triangles and lines, respectively) concentrations are shown. The ratio of particles per protein is depicted for sUC samples and for each fraction of UF-SEC isolated vesicles (**H**,**I**,**J**). In (**H**), values from all cell lines were pooled (*n* = 6). Values are presented as mean ± standard deviation. Friedman test with Dunn’s multiple comparison test, * *p* < 0.05, ** *p* < 0.01, *** *p* < 0.001.

As the first step in characterizing isolated vesicles, we determined the particle and protein concentrations among SEC fractions and sUC-isolated EVs (Figure 1). EV samples isolated from cell lines by sUC exhibited average particle concentrations ranging from 4.13 × 10^11^ to 6.03 × 10^11^ particles/mL and average protein concentrations from 1.29 to 4.06 µg/µL among the three cell lines (Figure 1A). EVs isolated from human plasma by sUC exhibited average particle concentrations of 0.95 × 10^11^ particles/mL for HD and 0.50 × 10^11^ particles/mL for NDMM, with average protein concentrations of 0.86 µg/µL for HD and 0.51 µg/µL for NDMM (Figure 1E). Due to the different volumes of initial conditioned media and plasma used for sUC and UF-SEC, the obtained EV samples exhibited quite distinct ranges of particle concentrations among isolation methods. To normalize particle concentrations to the initial sample volume, the yields of EV isolation were further calculated and compared in Section 2.2.

Analysis of SEC fractions showed similar profiles of particle and protein concentrations among all cell lines (Figure 1B–D). Fractions 1 and 2 exhibited the lowest particle concentrations, with average values ranging from 0 to 0.11 × 10^11^ particles/mL. Fraction 3 showed the highest particle concentration, with MM.1S, ANBL-6, and ALMC-1 presenting average concentrations of 1.36 × 10^11^, 1.16 × 10^11^, and 0.83 × 10^11^ particles/mL, respectively. Fraction 4 presented the second-highest particle concentration, with average values ranging from 0.52 × 10^11^ to 0.69 × 10^11^ particles/mL. Fraction 5 displayed an intermediate particle concentration between fractions 2 and 4, with values ranging from 0.21 × 10^11^ to 0.37 × 10^11^ particles/mL. When considering protein concentrations, fractions 1 and 2 exhibited similar values for all cell lines, ranging from 0.12 to 0.14 µg/µL. In fractions 3, 4, and 5, protein concentrations progressively increased across all cell lines, with average values ranging from 0.18 to 0.34 µg/µL. In EV samples isolated from human plasma, the profiles of particle and protein concentrations were slightly different between HD and NDMM (Figure 1F,G). The highest particle concentration was observed in fraction 4 for HD (8.00 ± 3.71 × 10^11^ particles/mL), and in fraction 3 for NDMM (11.35 ± 9.16 × 10^11^ particles/mL), followed by fractions 3 or 4, respectively, and then fractions 5, 2, and 1. The protein concentration progressively increased from fraction 1 to 5, reaching 0.55 ± 0.31 µg/µL in HD and 0.49 ± 0.11 µg/µL in NDMM in fraction 5.

The particle-to-protein ratio (Figure 1H–J) provides further insights into the level of protein contamination of EV samples among isolation methods and SEC fractions. In EVs isolated from cell lines, SEC F3 showed the highest ratio, with an average value of 5.69 ± 1.42 × 10^8^ particles/µg among the three cell lines, indicating the highest purity of all EV samples. sUC and SEC F4 samples presented decreased purity, with an average ratio for sUC of 2.44 ± 1.30 × 10^8^ particles/µg among cell lines. SEC F5 and F2 had significantly lower ratios than SEC F3, with average values of 0.97 ± 0.26 × 10^8^ particles/µg for F5 (*p* = 0.0026) and 0.66 ± 0.26 × 10^8^ particles/µg for F2 (*p* = 0.0006) among cell lines, indicating higher protein contamination in these fractions. In EVs isolated from human plasma, the ratios observed in SEC F3 were even higher, reaching average values of 45.18 ± 28.41 × 10^8^ particles/µg for HD and 65.76 ± 46.19 × 10^8^ particles/µg for NDMM, both significantly higher than sUC ratios (HD, 1.31 ± 0.67 × 10^8^ particles/µg, *p* = 0.0022; NDMM, 2.60 ± 3.26 × 10^8^ particles/µg, *p* = 0.0024).

In summary, in both EVs isolated from cell lines and from plasma, SEC fractions 3 and 4 contained the highest concentrations of particles, and fraction 3 presented the highest particle-to-protein ratio among UF-SEC fractions. Consequently, we focused on fraction 3 for subsequent analyses. In terms of protein concentrations, fraction 5 displayed the highest concentration, followed by fractions 4, 3, 2, and 1. Additionally, the comparison of the particle-to-protein ratio between the two isolation methods indicates that UF-SEC samples, particularly F3, exhibit higher EV purity than sUC samples.

#### 2.1.2. EV Size and Morphology

**Figure 2:**
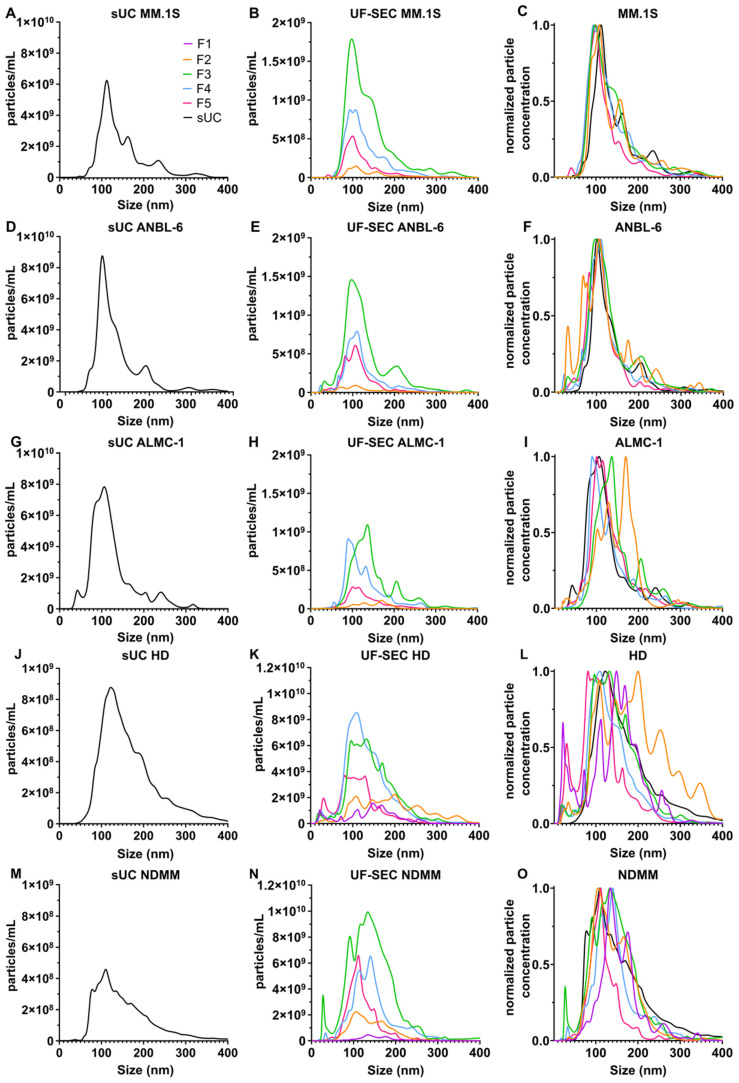
Concentration and size analysis of sUC- and UF-SEC-isolated EVs. Each curve represents the average of NTA curves of EV samples obtained from independent cell cultures of MM.1S ((**A**–**C**), *n* = 2), ANBL-6 ((**D**–**F**), *n* = 2), and ALMC-1 ((**G**–**I**), *n* = 2) cell lines and from plasma samples of HD ((**J**–**L**); *n* = 19 for sUC and *n* = 5 for UF-SEC) and NDMM patients ((**M**–**O**); *n* = 24 for sUC and *n* = 5 for UF-SEC). As sUC and UF-SEC samples contained distinct particle concentrations, curves in ((**A**,**B**,**D**,**E**,**G**,**H**,**J**,**K**,**M**,**N**)) were normalized by their maximum value (curves in (**C**,**F**,**I**,**L,O**)). For UF-SEC, fraction 1 (F1, purple curve), fraction 2 (F2, orange curve), fraction 3 (F3, green curve), fraction 4 (F4, blue curve), and fraction 5 (F5, pink curve) were analyzed. F1 was omitted for cell lines because of the undetectable particle concentration in this fraction. sUC curves are represented in black.

We assessed the size distribution of vesicles in sUC samples and SEC fractions. Figure 2 displays the average particle concentrations according to the size distribution. To facilitate the comparison of EV sizes between isolation methods, curves were further normalized by their maximum value (Figure 2C,F,I,L,O).

Among SEC fractions, modal size values ranged from 93.5 nm (F4) to 107.5 nm (F2) for MM.1S and from 97.5 nm (F2) to 110.5 nm (F4) for ANBL-6. For ALMC-1, fractions exhibited a wider range of modal sizes: 169.5 nm for F2, 136.5 nm for F3, 90.5 nm for F4, and 100.5 nm for F5. The modal size of HD EV samples ranged from 81.5 (F5) to 199.5 (F2) nm (Figure 2K) and from 105.5 (F2) to 140.5 (F4) nm in NDMM samples (Figure 2N).

Comparing the isolation methods, the modal size for sUC vs. UF-SEC F3 was 112.5 vs. 97.5 nm for MM.1S (Figure 2C), 101.5 vs. 97.5 nm for ANBL-6 (Figure 2F), 106.5 vs. 136.5 nm for ALMC-1 (Figure 2I), 122.5 vs. 132.5 nm for HD (Figure 2L), and 110.5 vs. 133.5 nm for NDMM (Figure 2O), respectively. Overall, all EV samples included mostly particles smaller than 400 nm and with a mode below 150 nm (except F2 from ALMC-1 and F2 from HD).

**Figure 3:**
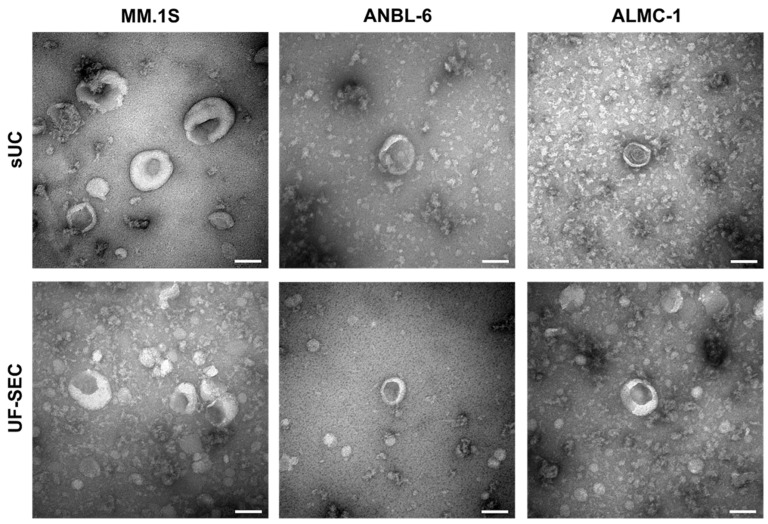
Transmission electron microscopy images of negatively stained EVs isolated by sUC and UF-SEC (fraction 3). Each image is representative of at least 10 fields from the same grid. Scale bars: 100 μm.

We used transmission electron microscopy to visualize the size and morphology of vesicular structures for the three cell lines and the two isolation methods (Figure 3). Vesicles from MM.1S, ANBL-6, and ALMC-1 cell lines exhibited similar shapes and sizes, as well as uniformity across both isolation methods. The observed morphology corresponds to the cup-shaped particles typically described in the literature, with sizes ranging approximately from 20 to 200 nm, consistent with the size distribution measured by NTA (Figure 2).

#### 2.1.3. Detection of EV Markers

**Figure 4:**
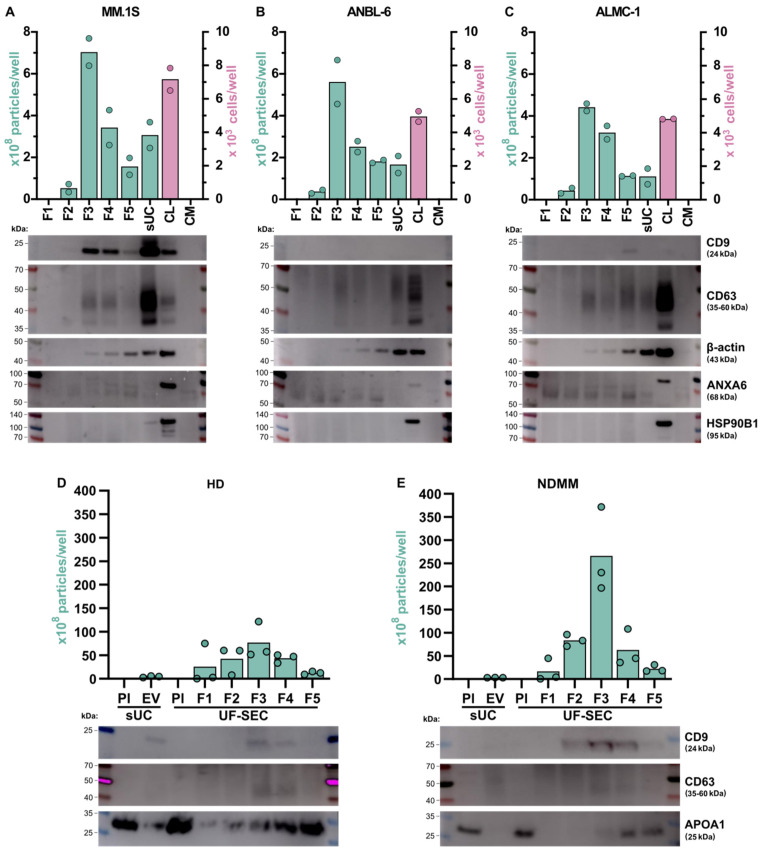
Detection of EV markers and contaminants. Western blot analyses were performed on EVs isolated from myeloma cell lines MM.1S (**A**), ANBL-6 (**B**), and ALMC-1 (**C**) and from plasma samples of HD (**D**) and NDMM patients (**E**). In each lane of the western blot on cell lines (**A**–**C**), 1 µg of protein was loaded per sample, and in each lane of the western blot on plasma samples (**D**,**E**), 2.5 µg of protein was loaded per sample. In UF-SEC isolated EVs, F1 to F5 fractions were included. The amount of EVs per well is represented on the left y axis of the graphics. Individual and mean values of the biological replicates are presented (green dots and bars, respectively). In (**A**–**C**), the number of cells present in each well of cell lysates (CL) is represented on the right y axis of the graphics. Individual and mean values of the biological replicates are presented (pink dots and bars, respectively). The negative control is complete culture medium (CM), either RPMI (for MM.1S) or IMDM (for ANBL-6 and ALMC-1) supplemented with EV-depleted FBS. In (**D**) and (**E**), the corresponding plasma (Pl) used for EV isolation was included. Analyzed EV markers included CD9 and CD63 (membrane markers), as well as β-actin and ANXA6 (cytosolic markers). A marker of large EVs, HSP90B1, was used for small EV specificity assessment in cell line samples. APOA1 was used as a non-EV contaminant marker for plasma samples. These images are representative of western blots reproduced on two independent cell cultures, three HD and three NDMM patients. Uncropped images can be found in Figure S1.

The presence of previously described EV markers [4] was determined by western blot analysis. These markers included the EV transmembrane tetraspanins CD9 and CD63, as well as the cytosolic proteins β-actin and ANXA6. APOA1 was used as a non-EV marker for plasma samples. The same amount of protein was loaded for all samples of the same polyacrylamide gel. The corresponding number of EVs or cells in each sample is graphically represented (Figure 4). For all tested EV markers, we observed no or very weak signals in fraction 1, which may be related to the low number of vesicles in these UF-SEC samples.

CD9 was clearly detected in MM.1S cells and derived EVs, with a strong signal in EVs isolated by sUC and a fainter but clear signal in F3, F4, and F5 of UF-SEC-isolated EVs. Conversely, little to no CD9 signal was detected in ANBL-6 and ALMC-1 cells and derived EVs. Regarding HD and NDMM samples, CD9 was mainly detected in fractions 3 and 4 of SEC and in sUC samples, although the signal was very weak in NDMM (Figure 4D,E).

CD63 was detected in EVs isolated by both techniques in all cell lines. For MM.1S, we observed a strong signal in EVs isolated by sUC and a light signal in F3, F4, and F5 of UF-SEC. For ANBL-6 and ALMC-1, the signal was mostly similar between EVs isolated by sUC and those in fractions 3, 4, and 5 of UF-SEC. Surprisingly, the CD63 signal was stronger in cell lysates than in EV samples for these cell lines. In EV samples from human plasma, the CD63 signal was barely visible in sUC samples, whereas in UF-SEC samples it was primarily detected at very low levels in fractions 3 and 4 from both HD and NDMM patients (Figure 4D,E).

**Figure 5:**
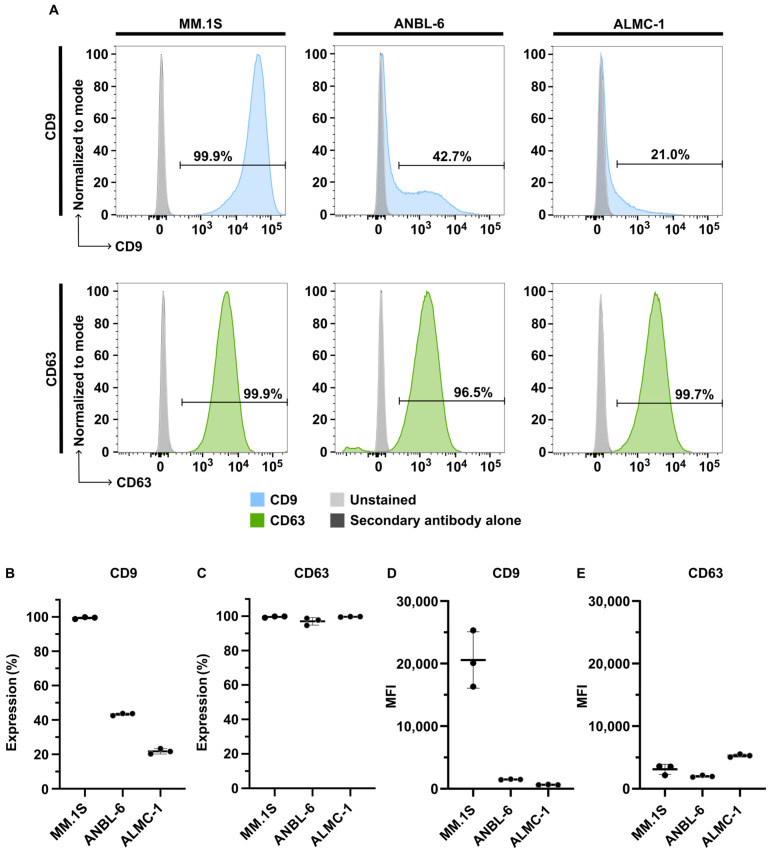
Flow cytometry analysis of CD9 and CD63 expression on live MM.1S, ANBL-6, and ALMC-1 whole cells. Representative histograms of CD9 (top; blue) and CD63 (bottom; green) expression by the MM.1S (left), ANBL-6 (middle), and ALMC-1 (right) cell lines (**A**). Percentage of expression of CD9 (**B**) and CD63 (**C**) by MM.1S, ANBL-6, and ALMC-1 cell lines. Mean fluorescence intensity (MFI) of CD9 (**D**) and CD63 (**E**) labeling on MM.1S, ANBL-6, and ALMC-1 cell lines. Analyses were repeated on three independent biological and three independent technical replicates. Negative controls were unstained (light grey) or incubated with secondary antibody alone (no primary antibody; dark grey). Only the unstained control is visible as both control curves are overlayed. In (**B**–**E**) values are presented as the mean of biological replicates ± standard deviation, and each dot corresponds to the mean of three technical replicates.

Given the differential expression of CD9 in EVs among the three myeloma cell lines, and the strong CD63 signal in cell lysates, we investigated whether the presence of CD9 and CD63 in EVs was correlated with their expression on the surface of the corresponding cell lines (Figure 5). Flow cytometry analysis revealed high CD9 expression in MM.1S (99.42 ± 0.56% of CD9-positive cells), intermediate expression in ANBL-6 (43.31 ± 0.73%), and low expression in ALMC-1 (21.78 ± 1.55%; Figure 5B). All three cell lines displayed substantial CD63 expression, with an average of 99.61 ± 0.38%, 97.06 ± 2.19%, and 99.69 ± 0.14% CD63-positive cells for MM.1S, ANBL-6, and ALMC-1, respectively (Figure 5C). Interestingly, the analysis of the mean fluorescence intensity (MFI) revealed a high level of CD9 at the surface of MM.1S cells, whereas ANBL-6 and ALMC-1 cells presented a much lower fluorescence intensity (Figure 5D). Regarding CD63, although a high proportion of the three cell types expressed this marker, the MFI was very low (Figure 5E). These results are consistent with the western blot observations. They suggest that the absence or reduced presence of CD9-positive EVs was associated with reduced marker expression at the cell surface, whereas positive CD9 signal in EVs correlated with strong expression of this marker at the cell surface. The pattern of expression of CD63 was consistent between the three cell lines, suggesting that this protein was expressed by all cells but at a low level, which might explain the weak signal in EV samples observed in the western blot membranes. The flow cytometry gating strategy can be found in Figure S2.

A robust β-actin signal was consistently detected in EV samples across all cell lines (Figure 4A–C). Interestingly, the signal intensity increased from F3 to F5 in EVs isolated by UF-SEC and was even stronger in sUC samples. This suggests that, unlike CD9 and CD63, where signal intensity in UF-SEC samples mostly correlated with the number of EVs, β-actin may be more associated with non-EV contaminant proteins. Regarding ANXA6, this marker was not detected in EV samples, although it was present in the cell lysates, suggesting that, in our conditions, EVs isolated from these myeloma cell lines do not carry this protein.

The HSP90B1 heat shock protein, also known as GRP94, is typically enriched in large EVs such as oncosomes or pathological/atypical EVs [4]. It was included in the western blot antibody panel to determine the presence of large EV contamination, and to assess the small EV specificity of isolated EV samples (Figure 4A–C). Our findings indicate that neither ANBL-6- nor ALMC-1-isolated EVs, whether through UF-SEC or sUC, express HSP90B1. However, MM.1S EV samples isolated by sUC exhibited a weak HSP90B1 band, suggesting the presence of some particles with large EV characteristics. This could indicate the existence of a slightly more heterogeneous population of EVs in the sUC-isolated samples than in EVs isolated by UF-SEC.

APOA1 is a plasma lipoprotein that can be co-isolated with EVs and is used as a marker of contamination in EV samples. The western blot analysis of EV samples isolated from plasma indicates that F1 to F5 show increasing contamination by APOA1 with sUC samples exhibiting contamination levels comparable to those of F2 or F3.

### 2.2. Yield and Reproducibility of EV Isolation by sUC vs. UF-SEC

**Figure 6:**
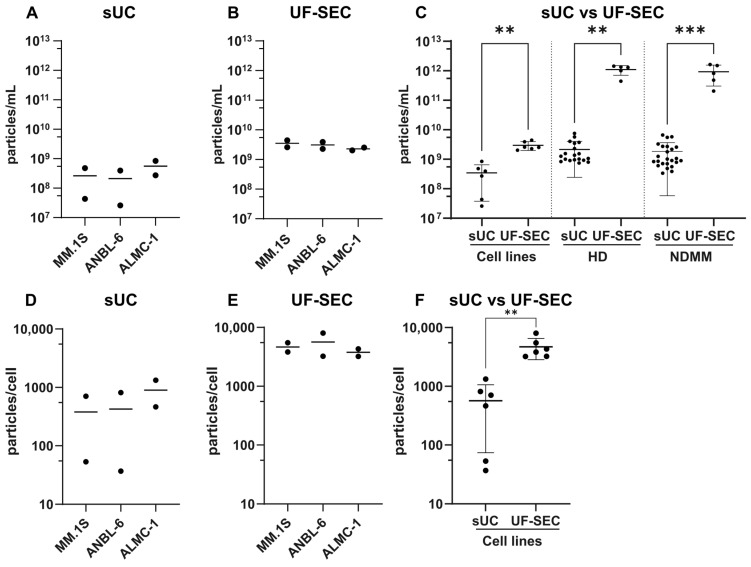
Yields of EV isolation by sUC and UF-SEC from MM.1S, ANBL-6, and ALMC-1 cell lines and plasma samples. Yield of EV isolation by sUC for individual cell lines, expressed in particles/mL (**A**). Yield of EV isolation by UF-SEC for individual cell lines, expressed in particles/mL (**B**). Yield of EV isolation by sUC vs. UF-SEC from cell lines and plasma samples of healthy donors (HD) and newly diagnosed multiple myeloma (NDMM) patients, expressed in particles/mL (**C**). Yield of EV isolation by sUC for individual cell lines, expressed in particles/cell (**D**). Yield of EV isolation by UF-SEC for individual cell lines, expressed in particles/cell (**E**). Yield of EV isolation by sUC vs. UF-SEC, for all cell lines, expressed in particles/cell (**F**). Yields are presented in particles/mL (total number of EVs isolated by sUC, or SEC fractions 1 to 5 divided by the initial volume of conditioned culture medium or plasma) and in particles/cell (total number of isolated EVs divided by the number of cells at the end of the medium conditioning). Values are presented as mean of two biological replicates (*n =* 2) in (**A**,**B**,**D**,**E**). In (**C**,**F**), values are presented as mean ± standard deviation. Kruskal–Wallis test (cell lines, *n* = 6; HD sUC, *n* = 19; HD UF-SEC, *n* = 5; NDMM sUC, *n* = 24; NDMM UF-SEC, *n* = 5), ** *p* < 0.01, *** *p* < 0.001.

To compare the efficiency and effectiveness of sUC and UF-SEC in isolating small EVs from conditioned cell culture medium and human plasma, we examined the yield and reproducibility of each method (Figure 6). For the cell lines, the yield of EV isolation was determined by dividing the total number of EVs recovered from fractions 1 to 5 by the initial volume of conditioned culture medium (Figure 6A–C) or by the number of cells at the end of the medium conditioning (Figure 6D–E). Both metrics were considered because ANBL-6 and ALMC-1 have lower plating densities than MM.1S cells, resulting in fewer cells in the same volume of CCM. For EVs isolated from plasma, the isolation yield was determined by dividing the total number of recovered EVs by the initial volume of plasma (Figure 6C).

Within each technique, we observed that the yield of EV isolation was similar among the cell lines (Figure 6A,B,D,E). Consequently, the results from MM.1S, ANBL-6, and ALMC-1 were pooled for analysis (Figure 6C,F). EV isolation yields were 3.44 ± 3.06 × 10^8^ particles/mL for sUC vs. 29.65 ± 9.78 × 10^8^ particles/mL for UF-SEC and 567.40 ± 493.30 particles/cell for sUC vs. 4679.00 ± 1848.00 particles/cell for UF-SEC. This means that UF-SEC produced a significantly higher EV isolation yield than sUC, being able to recover, on average, 8.63 times more EVs per volume of CCM (*p* = 0.0029) and 8.25 times more EVs per number of cells (*p* = 0.0022) compared to sUC. This difference in yield is even more significant when EVs are isolated from plasma samples. EV isolation yields were 21.46 ± 19.03 × 10^8^ particles/mL for sUC vs. 11 052.00 ± 4 006.81 × 10^8^ particles/mL for UF-SEC (*p* = 0.0083) in HD and 18.59 ± 18.01 × 10^8^ particles/mL for sUC vs. 9 318.00 ± 6 280.77 × 10^8^ particles/mL for UF-SEC (*p* = 0.0008) in NDMM, meaning that UF-SEC yields are 515.25 and 500.93 times higher than sUC yields for HD and NDMM samples, respectively.

In addition to yield, we compared the reproducibility between the two methods using the coefficient of variance (CV). CV is calculated as the standard deviation of one group of values divided by the mean of those values ×100. A low value indicates that the variability of the method is low. For cell lines, the coefficient of variance of the EV isolation yield expressed in particles/mL (Figure 6C) is 88.96% for sUC vs. 33.00% for UF-SEC. Similarly, when the yield is presented in terms of EV/cell (Figure 6F), the coefficient of variance is 86.95% for sUC vs. 39.49% for UF-SEC. Similar results were obtained for EVs isolated from plasma, as the CV for sUC is 88.69% and 96.91% vs. 36.31% and 67.39% for UF-SEC in HD and NDMM samples, respectively (Figure 6C). This indicates that, in the case of cell lines, the relative standard deviation of sUC is 2.70 times greater than that of UF-SEC for the particles/mL yield and 2.20 times greater for the particles/cell yield. The corresponding ratio for plasma samples is 2.44 and 1.44 for HD and NDMM groups, respectively. Overall, our findings show that UF-SEC enables EV isolation with higher yield and better reproducibility than sUC, for EVs from cell lines and plasma.

### 2.3. Comparison of Buffers for EV Recovery and Preservation

Although freshly isolated EVs offer optimal yield [24], they are typically stored at −80 °C in PBS [25]. However, other buffers have been suggested as advantageous compared to PBS regarding EVs’ physical stability and recovery rate. It has been previously shown that PBS supplemented with 0.005% Tween 20 (PBS-T) or HBS resulted in a better EV recovery rate and EV preservation than PBS alone [26]. However, the combination of HBS and Tween (HBS-T) was not tested.

**Figure 7:**
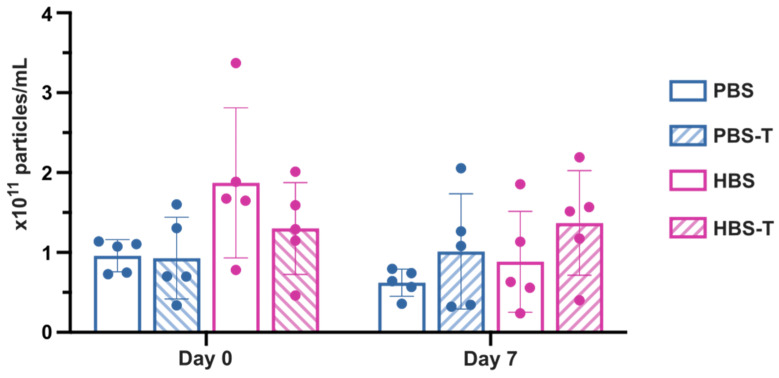
Comparative analysis of EV recovery and preservation upon −80 °C storage in different buffers. PBS, PBS + 0.005% Tween 20 (PBS-T), HBS, or HBS + 0.005% Tween 20 (HBS-T) buffers were used to elute EV samples from SEC columns. Particle concentrations were determined by NTA on fresh samples (day 0) and after 7 days of storage at −80 °C (day 7). Each replicate corresponds to an independent cell culture of the MM.1S cell line. Results are presented as mean values ± standard deviation. Kruskal–Wallis with Dunn’s multiple comparison test and Wilcoxon test with Bonferroni correction (*n* = 5).

Here, we compared the effect of PBS, PBS-T, HBS, and HBS-T on EV recovery and preservation (Figure 7). Using various elution buffers and the UF-SEC technique, we isolated MM.1S-derived EVs from conditioned medium. EV concentration was determined both immediately after EV recovery (day 0) and after 7 days of storage at −80 °C (day 7). This characterization was done in fraction 3, as it is the most enriched in particles. Our results suggest an improved EV recovery with HBS (1.87 ± 0.94 × 10^11^ particles/mL) compared to PBS (0.96 ± 0.20 × 10^11^ particles/mL), PBS-T (0.93 ± 0.51 × 10^11^ particles/mL), and HBS-T (1.30 ± 0.57 × 10^11^ particles/mL), although the difference between groups is not statistically significant (Kruskal–Wallis with Dunn’s multiple comparison test, *p* = 0.0841, *n* = 5). Furthermore, the presence of Tween 20 did not appear to influence the recovery rate, as we did not observe a discernible pattern in that regard.

Interestingly, after 7 days of storage at −80 °C, EV concentration slightly decreased in PBS and HBS (0.96 ± 0.20 × 10^11^ particles/mL (day 0) vs. 0.62 ± 0.17 × 10^11^ particles/mL (day 7), *p* = 0.0625, and 1.87 ± 0.94 × 10^11^ particles/mL (day 0) vs. 0.88 ± 0.63 × 10^11^ particles/mL (day 7), *p* = 0.0625, respectively, Wilcoxon test with Bonferroni correction), while remaining relatively stable in buffers containing Tween 20. Notably, HBS-T exhibited the highest EV concentration, with 1.37 ± 0.65 × 10^11^ particles/mL.

Taken together, these findings show a tendency towards improved EV recovery rates with HBS and enhanced EV preservation with HBS-T as the SEC eluent buffer. Consequently, we utilized HBS-T as the buffer for all EV isolations in this study, whether by UF-SEC or sUC.

## 3. Discussion

In this study, we addressed the challenge posed by the lack of a standardized EV isolation method by conducting a comparative analysis of two isolation techniques: sucrose cushion ultracentrifugation (sUC) and ultrafiltration combined with size-exclusion chromatography (UF-SEC). We evaluated their yield, reproducibility, and specificity for isolating small EVs while exploring various buffers for EV recovery and preservation. Isolating EVs using different methods from various multiple myeloma cell lines or human plasma may yield different subtypes of vesicles with varying characteristics. Hence, we characterized these vesicles by assessing protein and particle concentrations, particle size distribution, morphology, and expression of EV markers.

The characterization of isolated EVs by NTA revealed that the vesicle size distribution was similar in both isolation methods, as well as across various SEC fractions and in cell lines and plasma samples. The size distribution suggests that the isolated particles consisted mostly of exosomes and/or small ectosomes (30–150 nm), with some small microvesicles and/or apoptotic bodies (150–350 nm). Most particles ranged from 20 nm to 400 nm in both isolation methods, indicating minimal contamination by large EVs, such as oncosomes or apoptotic bodies. It is worth noting that non-vesicular particles, i.e., EV-like components that do not contain a lipid-bilayer membrane [4], such as lipoproteins, supermeres, exomeres, and viral particles, which fall within this size window, may also be present in the samples. Additionally, the transmission electron microscopy images confirmed the presence of vesicles with the expected morphology.

The presence of transmembrane EV markers, CD9 and/or CD63, confirmed the enrichment, in small EVs, of samples isolated by both techniques. However, the signal intensity did not consistently correlate with the respective EV amount, being mostly stronger in sUC samples compared to any fraction of UF-SEC in the case of EVs isolated from cell lines. This variation suggests a higher enrichment in CD9 and CD63 vesicles with sUC, especially in MM.1S. EVs isolated from human plasma presented CD9 expression, as well as a weak CD63 signal, which might reflect a more heterogeneous population of vesicles in plasma compared to those from a pure cell line. β-actin has been proposed as a cytosolic EV marker by MISEV 2018 guidelines [4] and is commonly used as a loading control for EV samples in western blots analysis. However, our findings indicate that the signal of β-actin is not correlated with either the quantity of EVs or with the protein amount. This suggests that, under our experimental conditions and for the specific cell lines used, β-actin may not be a suitable protein-loading control for EV samples or an appropriate cytosolic EV marker. ANXA6 is another described cytosolic EV marker. Although we were able to detect ANXA6 in myeloma cell lysates, no signal was observed in EV samples from both isolation techniques. This suggests that either the isolation methods employed in this study exclude vesicles containing ANXA6, or that these myeloma cells do not produce ANXA6-containing EVs. Such discrepancies can arise due to variations in cargo selection in EVs, influenced by factors such as cell type, microenvironment, and cell biology [27]. Despite its inclusion in MISEV guidelines [4], ANXA6 may be unsuitable as a small EV marker for myeloma cells under our experimental conditions. Finally, we examined the HSP90B1 marker to assess the contamination by large vesicles in EV samples. No signal was detected in UF-SEC samples, indicating the specificity of this method for isolating small EVs. However, a faint signal was observed in sUC-EV samples from MM.1S cells. While this suggests a less stringent isolation of small EVs by this technique, it is important to note that this observation was limited to only one cell line out of three.

The comparison of particle and protein concentrations between isolation methods revealed expected differences due to the varying volumes of conditioned culture medium and plasma utilized for sUC and UF-SEC. Among UF-SEC EV samples, fraction 3 exhibited the highest particle concentration, followed by F4 and F5. Fraction 1 showed a very low or undetectable particle concentration, suggesting it may be neglected for subsequent studies. Regarding protein concentration, there was a gradual increase from F1 to F5. The particle-to-protein ratio highlights SEC F3 as the purest fraction, with the highest ratio value, containing 2.34 times less protein contamination than sUC samples isolated from cell lines, as well as 34.46 and 25.30 times less protein contamination than sUC samples isolated from HD and NDMM plasma, respectively. F2 and F5 appeared as the most contaminated fractions when isolating EVs from cell lines, whereas sUC and F5 samples were the most contaminated within EVs isolated from plasma. Depending on the purity requirements of downstream studies, excluding these fractions may be advisable. These results emphasize the need to characterize each fraction individually when working with new conditions and the significance of selecting the appropriate fraction window for subsequent studies, according to the initial source of EVs.

Because of the variability in the initial volumes used for EV isolation, we compared the efficacy of EV isolation for each technique by analyzing their yield and reproducibility. Our findings show that UF combined with SEC yields over 8 times more EVs from cell lines compared to sUC, and over 500 times more EVs from plasma, while ensuring greater reproducibility over replicates. In other words, the same initial volume of conditioned medium produces 8 or 500 times more EVs with UF-SEC than with sUC. Other advantages that are worth mentioning are that UF-SEC significantly reduces the isolation time compared to sUC (approximately 3 h vs. 26 h in our conditions), does not require specialized and expensive equipment like an ultracentrifuge (making it a more cost-effective technique), and its simple protocol requires less expertise to execute [12].

In this study, we compared EVs derived from three human myeloma cell lines: MM.1S, ANBL-6, and ALMC-1, and from HD and NDMM patients’ peripheral blood. Our results revealed similar particle concentrations and size distributions among EVs isolated from the three cell lines and from plasma, with most particles ranging from 20 nm to 400 nm. The most notable variation among the cell lines was observed in the expression of EV markers. While CD63 was detected in EV samples from all cell lines, CD9 was predominantly observed in MM.1S-derived EVs. These findings were further supported by flow cytometry analysis, showing that the presence of CD63 and CD9 in EVs was related to the level of expression at the surface of the cells. The recent literature suggests that these proteins, widely accepted as EV markers, are associated with distinct EV subtypes: exosomes for CD63 and small ectosomes for CD9 [28]. Based on our findings, we propose that MM.1S cells produce both CD9-positive ectosomes and CD63-positive exosomes, while ANBL-6 and ALMC-1 primarily secrete CD63-positive exosomes with minimal CD9-positive ectosome production.

Optimizing EV isolation is a key challenge, often due to inadequate buffers that cause EV aggregation and degradation, altering their structure and function [29,30]. Our study explored various recovery and preservation buffers, suggesting that HBS may yield more freshly isolated particles than PBS. While Tween 20 may not enhance EV recovery, it shows potential for improving EV preservation during storage at −80 °C. Combining HBS with Tween 20 appears to reduce particle loss over 7 days. Tween 20 has been previously described to coat solid–liquid interfaces [26], enhancing EV isolation efficacy. These results emphasize the importance of selecting suitable storage buffers to maintain EV integrity and functionality throughout isolation and storage processes.

In conclusion, our results highlight UF-SEC as an advantageous EV isolation method over sUC, giving better results in terms of yield, reproducibility, and purity of EV production, while being less time-consuming. The comparison of three myeloma cell lines, MM.1S, ANBL-6, and ALMC-1, revealed distinct expression of EV biomarkers, emphasizing the importance of cell line selection in EV research. Interestingly, the increased purity and yield in EV isolation observed with UF-SEC in comparison with sUC were much higher when using plasma than when using cell lines as the source of EVs. This indicates that UF-SEC is particularly indicated for the processing of EVs from human plasma, which is of great relevance for potential clinical applications. Finally, we suggest that the combination of HBS and Tween 20 may improve EV recovery and preservation over the traditional PBS, highlighting the need for further optimization of EV storage conditions.

This study has advanced the characterization of existing EV isolation methods and myeloma cellular models. The identification of UF-SEC as a more reproducible technique marks a step towards enhanced standardization in EV isolation. Given the wide-ranging implications of isolation standardization for both fundamental EV research and its clinical applications, it is of critical importance to continue research in this area.

## 4. Materials and Methods

### 4.1. Cell Culture

The human MM cell line MM.1S was purchased from ATCC (Manassas, VA, USA). MM.1S cells were cultured in RPMI 1640 medium (Corning, Corning, NY, USA) supplemented with 10% fetal bovine serum (FBS, Gibco, Waltham, MA, USA) and 1% penicillin-streptomycin (Corning) in 175 cm^2^ Nunclon Delta surface-treated EasYFlasks (Thermo Scientific, Waltham, MA, USA). Human MM cell lines ANBL-6 and ALMC-1 were purchased from Merck (Rahway, NJ, USA). These cell lines were cultured in Iscove’s Modified Dulbecco’s Medium (IMDM, Gibco) supplemented with 10% FBS, 1% penicillin-streptomycin, 1 ng/mL IL-6 (Gibco), and 2 mM L-glutamine (Gibco). All cells were maintained in an incubator at 37 °C and 5% CO_2_, and culture media were renewed every 2 or 3 days.

### 4.2. Culture Medium Conditioning

EVs were isolated from the conditioned culture medium (CCM), collected after 72 h of cell culture in a complete medium with EV-depleted FBS. The latter was obtained after FBS ultracentrifugation at 100,000× *g* for 140 min at 10 °C. For CCM production, between 1.8 × 10^8^ and 3.0 × 10^8^ cells were cultured in 577.7 ± 110.7 mL, depending on the cell line. MM.1S cells were plated at 5.0 × 10^5^ cells/mL, while ANBL-6 and ALMC-1 cells were plated at 3.0 × 10^5^ cells/mL. After 72 h, cells were harvested, centrifuged at 290× *g* for 5 min at room temperature (RT), and washed twice in cold phosphate-buffered saline (PBS, Corning). Cells were counted and lysed for western blot analysis. CCM was centrifuged at 500× *g* for 10 min and then at 3000× *g* for 20 min at 4 °C. Supernatants were stored at −80 °C for further EV isolation.

### 4.3. Patients’ Inclusion and Plasma Samples Collection

Healthy donors (HD, n = 19) and newly diagnosed multiple myeloma (NDMM, n = 24) patients were included from May 2016 to July 2020 after study approvals by ethical committees of the involved institutions, and human samples were collected after signed informed consent. All procedures followed the Helsinki Declaration, and participants gave written informed consent before study inclusion. Peripheral blood (PB) samples were collected prospectively according to the clinical follow-up schedule into separate EDTA tubes, with up to 18 mL of total PB collected. Tubes were stored at 4 °C in the upright position until centrifugation on the same day (<12 h) at 500 g for 10 min. The collected plasma supernatants were centrifuged at 3000× *g* for 20 min to eliminate cellular debris and apoptotic bodies and were stored at −80 °C until further EV isolation.

### 4.4. EV Isolation by Ultrafiltration Followed by Size-Exclusion Chromatography

For EV isolation by UF-SEC, 15 mL of CCM and 0.73 mL of plasma were used for each sample. First, an ultrafiltration step was performed using Amicon Ultra 15 centrifugal concentrators with Ultracel 100 kDa molecular weight cutoff filters (Merck). After centrifugation at 4000× *g* for 60 min at RT, a concentrated volume of 160 to 345 µL was recovered. From this, 150 µL was used for size-exclusion chromatography using qEVsingle Gen2 35 nm columns (Izon Science, Christchurch, New Zealand). Five separate fractions of 170 µL each were recovered, as recommended by the manufacturer. Different buffers were used as eluents (PBS, PBS + 0.005% Tween 20 (PBS-T), HEPES buffered saline (HBS; 5.96 g/L of HEPES, 9 g/L of sodium chloride), or HBS + 0.005% Tween 20 (HBS-T)). Recovered fractions were either used immediately for EV characterization by NTA or stored at −80 °C until further use.

### 4.5. EV Isolation by Sucrose Cushion Ultracentrifugation

EVs were isolated by sequential ultracentrifugation using a 30% sucrose cushion (sUC), as previously described [31], with slight modifications. All centrifugations were performed at 10 °C using the Optima XPN-100 ultracentrifuge (Beckman Coulter, Brea, CA, USA) with 45Ti or 70Ti rotors.

For EV isolation from cell lines, an average volume of 500.50 ± 91.94 mL of CCM was used for each sample. For EV isolation from human plasma, an average volume of 5.21 ± 2.72 mL was used per sample. Thawed samples were centrifuged at 12,000× *g* for 20 min. The supernatant was centrifuged at 100,000× *g* for 2 h and 20 min, and the pellet was resuspended in 15 mL of filtered PBS. Subsequently, the sample was carefully placed on top of 4 mL of filtered sucrose solution in deuterium oxide (D_2_O, 300 g/L of protease-free sucrose, 24 g/L of Tris, pH = 7.4) and centrifuged at 100,000× *g* for 1 h and 10 min. The resulting bottom fraction was aspirated, supplemented with 16 mL of filtered PBS, and centrifuged at 100,000× *g* for a minimum of 16 h. The isolated vesicles were resuspended in 200 or 500 µL of PBS or HBS-T buffer, and stored at −80 °C until further use.

### 4.6. Nanoparticle Tracking Analysis

The concentration and size distribution of EVs were determined using the NanoSight NS300 nanoparticle tracking analysis system (Malvern Panalytical Ltd., Malvern, UK) following the manufacturer’s guidelines. The NTA instrument settings were set at camera levels ranging from 14 to 16 and a detection threshold of 5. EV samples were pre-diluted from 1:15 to 1:2000 in 0.22 μm filtered PBS, and five separate 30-s videos were recorded at RT. The captured data were processed using the NanoSight 3.4 software (Malvern Panalytical Ltd.).

### 4.7. Bicinchoninic Acid Protein Assay

For further western blot analyses, the protein concentrations of lysed cell pellets and EV samples were determined using a bicinchoninic acid assay (BCA) Kit (#BCA1, Sigma-Aldrich, St. Louis, MO, USA) following the manufacturer’s instructions. The absorbance at 562 nm was measured using the SPARK multimode microplate reader (Tecan Group Ltd., Männedorf, Switzerland). For this purpose, cell pellets and EV samples were lysed in cold RIPA buffer (1.5 g/L of glycine, 0.1 g/L of SDS, 0.1% (*v*/*v*) of Tween 20, pH = 2.2) supplemented with protease and phosphatase inhibitor EDTA-free (Thermo Scientific) and kept at 4 °C for 30 min. After this time, tubes were sonicated at 4 °C for 5 × 30 s and kept at this temperature for 15 min with agitation. In the case of cell pellets, centrifugation at 14,000× *g* for 15 min at 4 °C was performed, and the supernatants were recovered.

### 4.8. Western Blot

Western blot analysis was utilized to detect specific proteins, selected according to MISEV guidelines, in EV samples and cell pellets. Bolt LDS sample buffer (Invitrogen, Waltham, MA, USA) was used as a loading buffer. As a negative control for EVs derived from cell lines, complete culture medium (CM) with EV-depleted FBS, either RPMI (for MM.1S) or IMDM (for ANBL-6 and ALMC-1), was employed. Samples under reducing conditions were incubated in Bolt sample reducing agent (Invitrogen) and heated at 70 °C for 10 min. Electrophoresis was conducted using a Mini Gel tank (Invitrogen) filled with Bolt 2-MES SDS running buffer (Invitrogen). For each sample, 1 or 2.5 µg of protein was loaded per well onto a 15-well Bolt Bis-Tris 1.0 mm Mini Protein Gel 4−12% (Invitrogen). The same amount of protein was loaded for all samples on the same gel. Proteins were separated at a constant voltage of 180 V for 24 min, together with a spectra multicolor broad-range protein ladder (Thermo Scientific) as a molecular weight marker.

Subsequently, gels were rinsed in water and proteins were transferred to PVDF mini stacks (Invitrogen) using an iBlot 2 device (P0 transfer program, Invitrogen). PVDF membranes were rinsed in tris-buffered saline (TBS; 2.4 g/L Tris, 8.8 g/L NaCl, pH = 7.6) and blocked in SuperBlock blocking TBS buffer (Thermo Scientific) with 0.1% Tween 20 for 1 h at RT. Membranes were then incubated overnight at 4 °C with primary antibodies (Table 1). Following three washes in TBS + 0.1% Tween 20 (TBS-T) for 10 min, membranes were incubated with horseradish peroxidase (HRP)-conjugated secondary antibodies, either Goat anti-Mouse (Invitrogen #32430, 1:3000) or Goat anti-Rabbit (Invitrogen #32460, 1:5000) for 90 min at RT. Membranes were subsequently washed twice in TBS-T for 10 min, once in TBS for 10 min, and incubated in SuperSignal West Femto Maximum Sensitivity Substrate (Thermo Scientific) for 5 min according to the manufacturer’s protocol. Chemiluminescent signals were detected, and images were acquired using the Amersham Imager 600 (GE Healthcare, Chicago, IL, USA). Uncropped pictures of western blot membranes are provided in Figure S1.

### 4.9. Flow Cytometry

Around 1.0 × 10^6^ MM.1S, ANBL-6 and ALMC-1 cells were plated per well and treated with Fc block (Human TruStain FcX, BioLegend, San Diego, CA, USA) for 10 min at RT. Cells were incubated in darkness with a Live/dead Fixable Aqua Dead Cell Stain Kit (Invitrogen, 1:2000) for 20 min at 4 °C. Next, cells were washed and incubated in darkness with the primary antibodies CD9 (1:100) or CD63 (1:50) for 30 min at 4 °C. After washing, cells were incubated in darkness with the secondary antibody PE anti-mouse IgG (BioLegend, #406607, clone RMG1-1, 1:200) for 30 min at 4 °C. Finally, cells were washed twice, resuspended in FACS buffer (PBS, 2% (*v*/*v*) FBS inactivated, 0.75 g/L of EDTA), and transferred to flow cytometry tubes. Samples were acquired on the LSRFortessaTM X-20 (BD, Franklin Lakes, NJ, USA), and data were analyzed using FlowJo Software v10.9.0 (BD). Unstained and no primary antibodies were used as controls. The cell gating strategy is described in Figure S2.

### 4.10. Transmission Electron Microscopy

Transmission electron microscopy was performed at *Instituto Gulbenkian de Ciência*, Oeiras, Portugal. For this purpose, 3 µL of each sample in HBS-T, either sUC-isolated EVs or fraction 3 of UF-SEC, was absorbed for 20 min into M100 formvar copper grids coated with carbon and glow-discharged (30 mA for 30 s) using a Q150T ES Carbon Coater (Quorum Technologies, Lewes, UK). After washing, grids were negatively stained for 5 min using filtered 2% uranyl acetate, and blotting was performed to remove the excess. Samples were visualized on a Tecnai G2 Spirit BioTWIN transmission electron microscope (FEI Company, Hillsboro, OR, USA) operated at 120 kV, and images were captured using an Olympus-SIS Veleta CCD Camera.

### 4.11. Statistical Analysis

Statistical analyses were performed using the GraphPad Prism 9.5.1 software (San Diego, CA, USA) for all experiments with more than 3 biological replicates. All graphs were generated using this software. Results were defined statistically significant when *p* ≤ 0.05. Due to the small sample size, non-parametric tests were utilized. Detailed descriptions of all statistical analyses can be found in the figure legends corresponding to the respective experiments, and results are expressed as mean ± standard deviation.

### 4.12. EV-TRACK

The data presented in this study have been submitted to the EV-TRACK [32] knowledgebase and are accessible at https://evtrack.org/review.php using the EV-TRACK ID: EV240028.

## Figures and Tables

**Table 1:** Primary antibodies used for western blot and flow cytometry.

Antigen	Clone	Host	Supplier	Reference	Dilution used	Electrophoresis Condition
CD9	Ts9	Mouse	Invitrogen	10626D	1:1000	Non-reducing
CD63	Ts63	Mouse	Invitrogen	10628D	1:250	Non-reducing
β-actin	AC-15	Mouse	Invitrogen	MA1-91399	1:8000	Reducing
ANXA6	ns	Rabbit	Novus Biologicals	NBP1-90149	1:1000	Reducing
HSP90B1	4G7C7	Mouse	Proteintech	60012-2-Ig	1:5000	Reducing
ApoA1	532	Mouse	Invitrogen	MA5-14732	1:500	Reducing

ns, not specified by the vendor.

## Data Availability

The raw data supporting the findings presented in this study can be obtained from the corresponding author, upon reasonable request.

## Supplementary Materials

The following supporting information can be downloaded at: https://www.mdpi.com/article/10.3390/ijms25158496/s1.

## Abbreviations

BCA	Bicinchoninic acid protein assay
CCM	Conditioned culture medium
**CL**	Cell lysates
CM	Culture medium
EDTA	Ethylenediaminetetraacetic acid
EV	Extracellular vesicle
F	fraction
FACS	Fluorescence-activated cell sorting
FBS	Fetal bovine serum
HBS	HEPES buffered saline
HBS-T	HBS + 0.005% Tween 20
HD	Healthy donor
HEPES	4-(2-hydroxyethyl)-1-piperazineethanesulfonic acid
HRP	Horseradish peroxidase
IMDM	Iscove’s Modified Dulbecco’s Medium
LDS	Lithium dodecyl sulfate
MES	2-(N-morpholino) ethane sulfonic acid
MISEV	Minimal information for studies of extracellular vesicles
MM	Multiple myeloma
NDMM	Newly diagnosed Multiple Myeloma
NTA	Nanoparticle tracking analysis
*p*	*p*-value
PBS	Phosphate-buffered saline
PBS-T	PBS + 0.005% Tween 20
PVDF	Polyvinylidene difluoride
RIPA	Radioimmunoprecipitation assay
RT	Room temperature
SDS	Sodium dodecyl sulfate
SEC	Size-exclusion chromatography
sUC	Sucrose cushion ultracentrifugation
TBS	Tris-buffered saline
TBS-T	TBS + 0.1% Tween 20
UC	Ultracentrifugation
UF	Ultrafiltration
UF-SEC	Ultrafiltration combined with size-exclusion chromatography
