# Supplementary figures and images for: Size-Exclusion Chromatography: A Path to Higher Yield and Reproducibility Compared to Sucrose Cushion Ultracentrifugation for Extracellular Vesicle Isolation in Multiple Myeloma
Source: Int J Mol Sci. 2024 Aug 3;25(15):8496. doi: 10.3390/ijms25158496 (PMC11313515; doi:10.3390/ijms25158496)

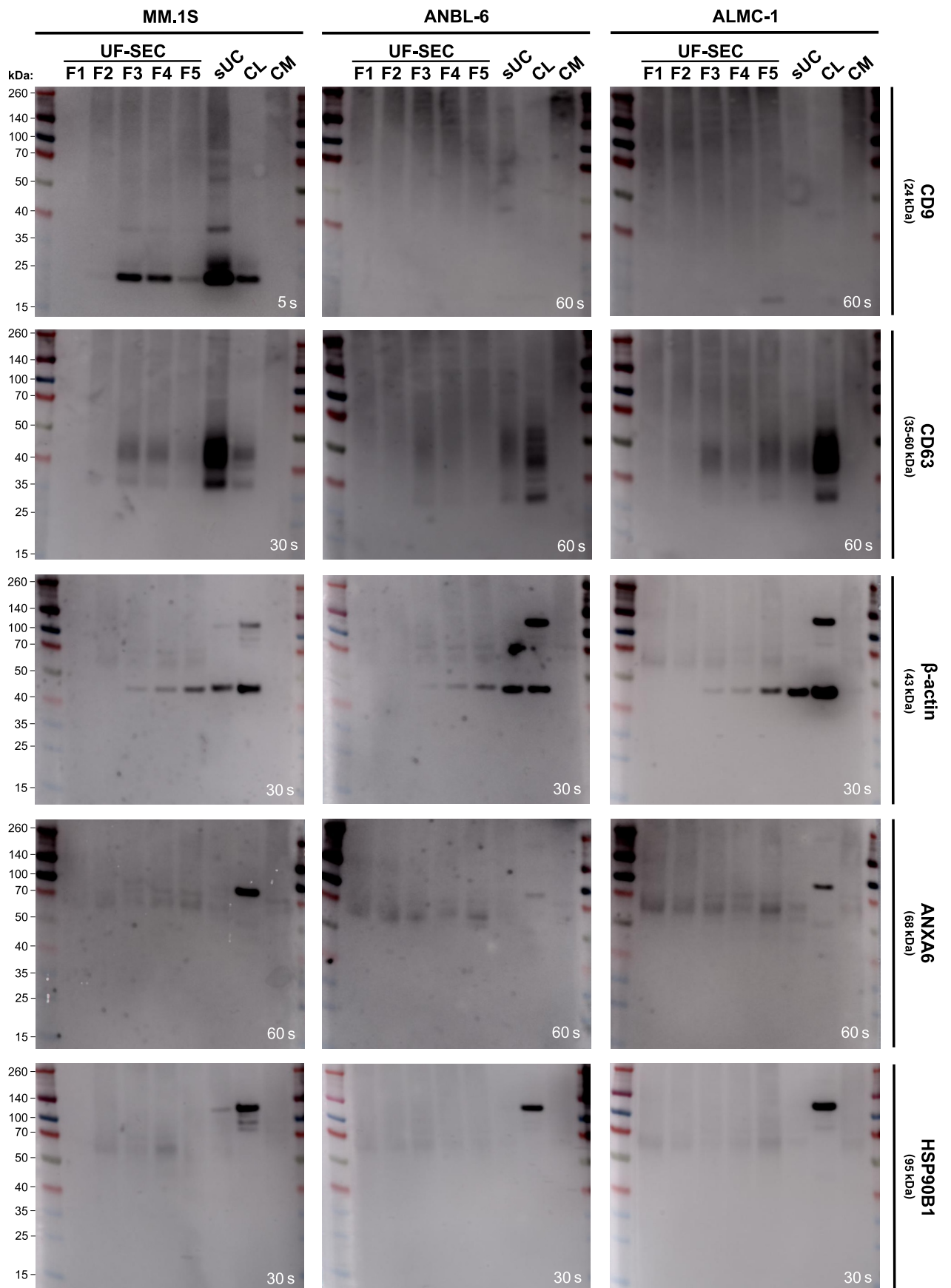

Supplement: Supplementary file 1 [file ijms-25-08496-s001.zip › Figure S1 p1.pdf]

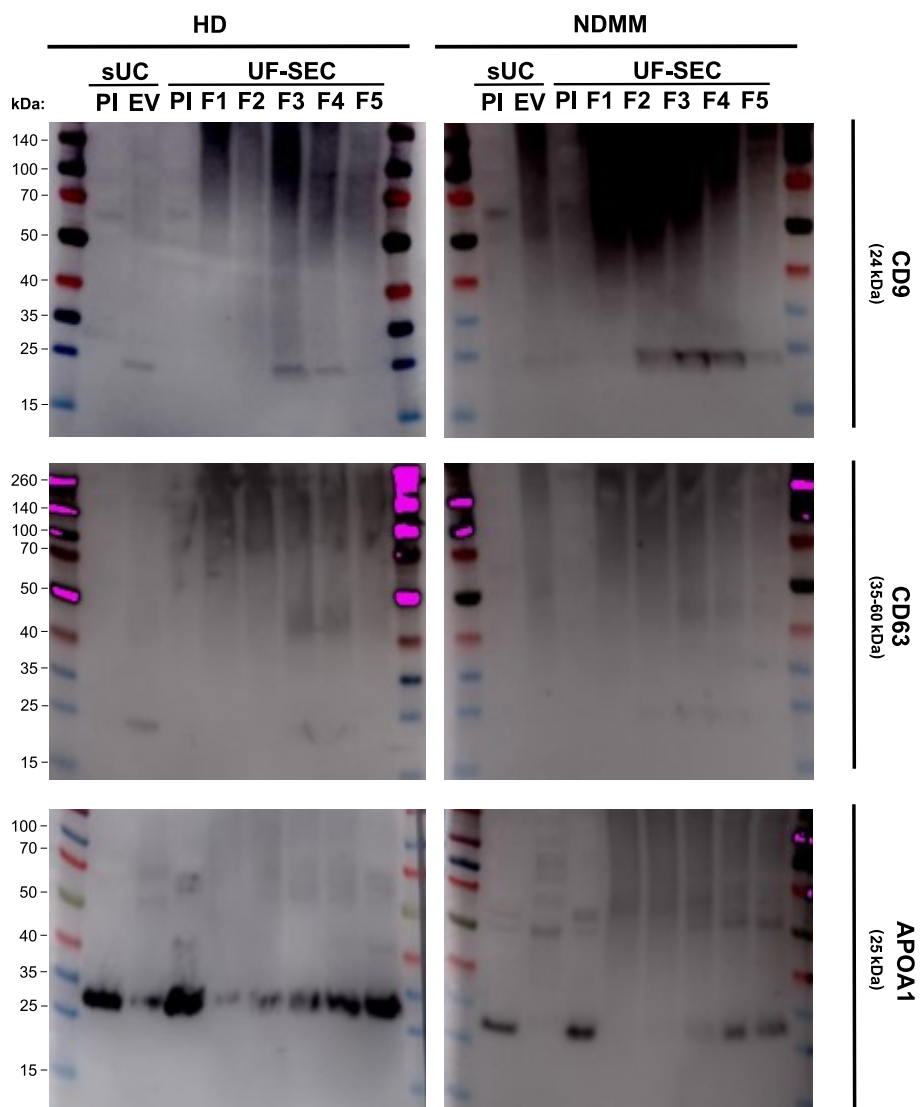

Supplement: Supplementary file 1 [file ijms-25-08496-s001.zip › Figure S1 p2.pdf]

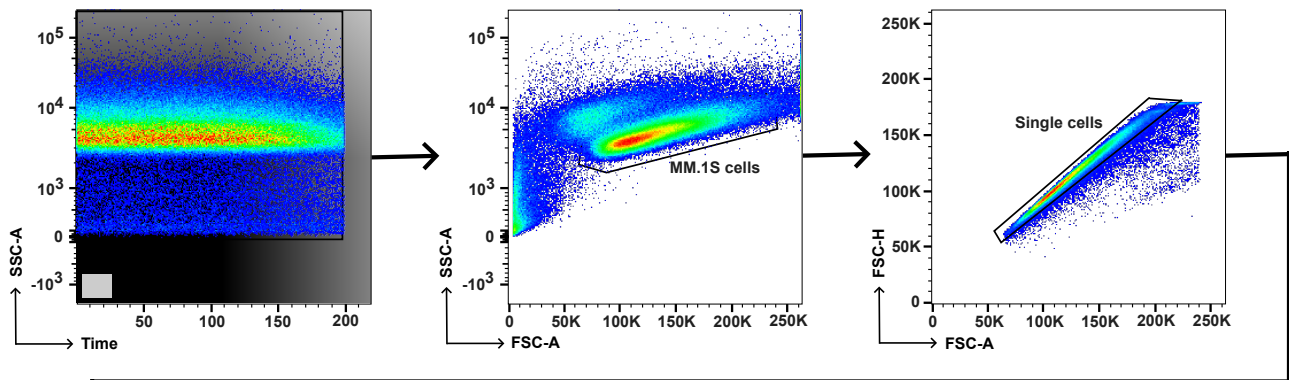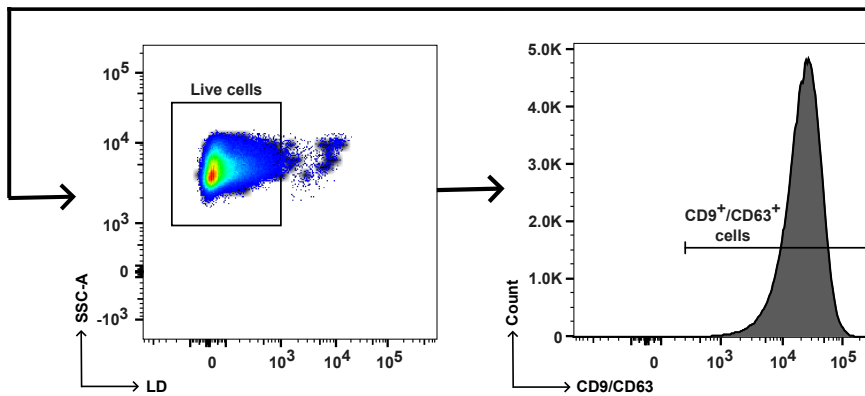

Supplement: Supplementary file 1 [file ijms-25-08496-s001.zip › Figure S2.pdf]
